# 

**Published:** 1887-12

**Authors:** 


					﻿DEOE1ZEBKR, 1887-
OLD SERIES
Vol. XXV
<^85?^?
NEW SERIES/
Vol. IV.—N*. IOX
Mm®
B JOUBNAEg.

©Wb,1
k W.F.WESTMOREtAND. IvW
H.V.M.M1LLER. M.D.LL .D.>£
JAMES A.GRAY. M.D.^
d/fflUSHED BY JA^SRHA^isON&.Ca|
Atlanta .ga- Jh
SUBSCRIPTION: *2.80 A YEAR.
				

